# How Thick Is the Oral Mucosa around Implants after Augmentation with Different Materials: A Systematic Review of the Effectiveness of Substitute Matrices in Comparison to Connective Tissue Grafts

**DOI:** 10.3390/ijms21145043

**Published:** 2020-07-17

**Authors:** Martin Lissek, Martin Boeker, Arndt Happe

**Affiliations:** 1 Institute of Medical Biometry and Statistics, Medical Data Science, Faculty of Medicine and Medical Center, University of Freiburg, 79104 Freiburg, Germany; martin.boeker@imbi.uni-freiburg.de; 2 Private Practice, Münster, Germany and Department of Oral and Maxillofacial Plastic Surgery and Implantology, University of Cologne, 50932 Köln, Germany; a.happe@dr-happe.de

**Keywords:** dental implantation, endosseous, tissue transplantation, soft tissue thickness, biomaterials, regenerative medicine

## Abstract

This systematic review aimed to assess the effectiveness of xenogeneic collagen matrices (XCMs) and acellular dermal matrices (ADMs) in comparison to connective tissue grafts (CTGs) for the augmentation of oral mucosa around dental implants. MEDLINE and the Web of Science were searched for clinical studies that compared substitute materials for the augmentation of oral mucosa to the subepithelial connective tissue graft around dental implants during or after implantation. The review was conducted according to the recommendations of the PRISMA statement. From an initial search result set of 1050 references, seven articles were included in the review. The study designs were heterogeneous, so no meta-analysis could be performed. Both the CTG and either type of substitute material resulted in increased mucosal thickness. Four studies showed no significant difference, while three demonstrated a significant difference, favoring the CTGs over alternative materials. Soft tissue augmentation around dental implants is a safe procedure and leads to thicker mucosal tissue. The subepithelial connective tissue graft can still be regarded as the gold standard, but substitute materials may be an acceptable alternative in some situations, such as for pain-sensitive patients, among inexperienced surgeons, and for sites with an already thick biotype.

## 1. Introduction

Basic animal studies dating back to 1996 have shown that soft tissue thickness around dental implants affects crestal bone remodeling after second stage surgery, as the biologic width establishes [1]. In this experiment, a thin biotype was artificially generated in a test group at the time of abutment connection. Although the length of the junctional epithelium hardly differed between the groups, the test group showed a continuous initial remodeling that led to bone loss. In a clinical study conducted by Linkevicius et al., patients with thick mucosa, for which the lower limit was set as 2 mm, showed significantly less post restorative remodeling than patients with thin mucosa [2].

But how exactly are a thick and a thin biotype actually defined? Olsson and Lindhe were the first to use the term biotype [3]. In their study published in 1991, they examined the periodontal conditions (probing, attachment level and amount of gingival recession) of the central maxillary incisors of 113 subjects. In addition, they calculated the ratio of crown width to crown length (CW:CL ratio). Subjects with longer, narrower incisors suffered significantly more often from recessions and had increased probing attachment levels. The authors henceforth referred to the biotype of patients with long narrow teeth as thin. Patients with rather wide and short central incisors were classified as thick biotype. These results confirmed previous animal studies which showed that plaque accumulation around tissues with low connective tissue density can lead to recessions [4,5]. The results of these studies could later be observed to a similar extent around implants. Patients with thin tissue showed a stronger tendency to recessions around implants [6]; so a thick biotype was defined as one of the success factors in implantology [7]. It should be noted that Olsson and Lindhe in their study neither examined the thickness of the mucosa nor did they define a threshold from which one can speak of a thick or thin type.

As described mucosal thickness has been identified as a factor influencing implant success [8]. Already in the early 1970s there were; therefore, efforts to thicken the oral soft tissue around natural teeth using connective tissue grafts [9]. Techniques to improve the gingival situation around implants have been developed and tested [10]. Connective tissue grafts from the lateral palate or tuber represent the gold standard to increase the thickness of the mucosa and can significantly influence the esthetic final result as well as the amount of marginal bone loss [11].

At the same time, patients and dentists are increasingly concerned with the overall esthetic appearance of implant restorations [8]. Particularly in the anterior region, “pink aesthetics” have gained more and more importance in recent years and specific scores like the Pink Esthetic Score [9,10] are currently used to evaluate esthetic appearance in clinical studies. Führhauser reported that 60% of his patients showed a greyish appearance of the peri-implant mucosa due to shine-through effects of restorative materials. These shine-through effects also seem to be related to the thickness of the soft tissue [11].

In the last few years collagen biomaterials as substitutes for autogenous connective tissue have been introduced [12,13]. These materials are mainly acellular matrices derived from xenogeneic or allogenic dermis and have several advantages including that the material is available almost indefinitely, a second surgical site can be avoided and the overall treatment time can be shortened. The harvesting of subepithelial connective tissue grafts is a demanding surgical technique and associated with considerable intra- and postoperative risks of bleeding, infection or necrosis [14].

Gargallo-Albiol et al. [15] published a meta-analysis comparing xenogeneic collagen matrices to the subepithelial connective tissue graft in 2019. The workgroup included studies that were published until 2018. The present paper offers new and longer-term data, more included trials and differs in some observations as well as the conclusion from the work mentioned above. We believe that it can only be advantageous if there are several references for scientists in the field. We hope to clearly state differing results and interpretations between this and the previous work. Therefore, the objective of this systematic review is to assess the effectiveness of substitute biomaterials in comparison to connective tissue grafts (CTGs) for the augmentation of oral mucosa around dental implants. It includes randomized trials that examine the medium- and long-term results that can be expected regarding augmentation of the buccal mucosa with the so called “gold standard” subepithelial connective tissue graft in comparison to substitute materials, thereby providing practitioners with information to guide evidence-based decisions on the use of these new biomaterials.

## 2. Results

### 2.1. Study Selection

**Figure 1:**
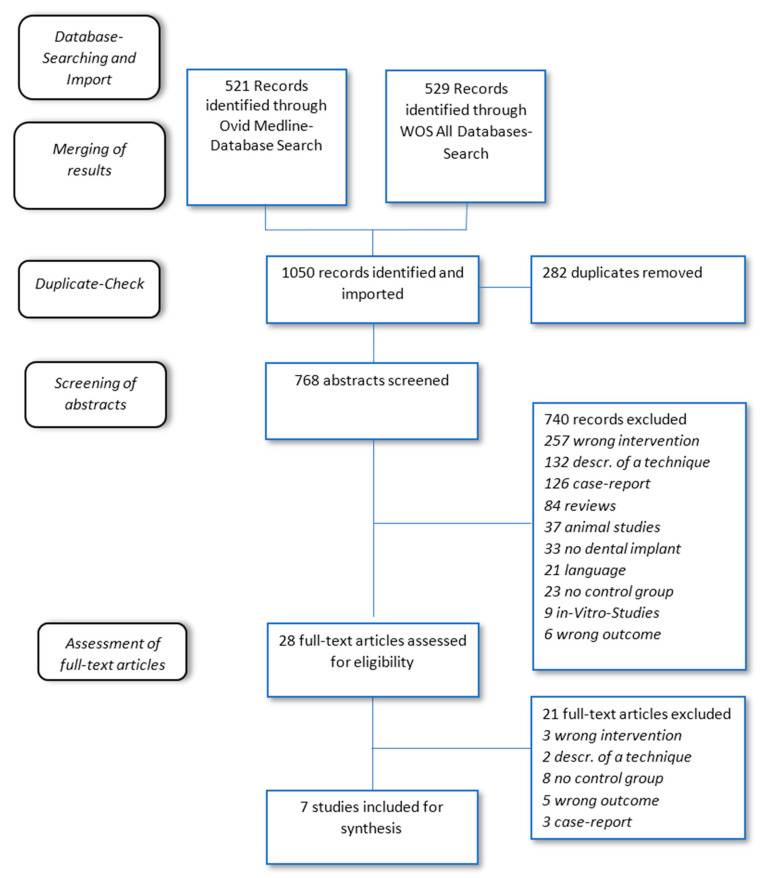
Study selection process.

Seven studies [16,17,18,19,20,21,22] fulfilled the inclusion criteria, two of those were classified as Follow-ups [17,19]. We identified 1050 potentially relevant studies through a systematic electronic database search (521 Ovid Medline + 529 WoS All Databases). After removal of duplicates 768 studies remained for screening. 28 full-text-articles were assessed and evaluated, of which 21 were excluded (Figure 1). The exclusion of each relevant study is justified in the additional materials.

### 2.2. Thickness of Oral Mucosa

**Figure 2:**
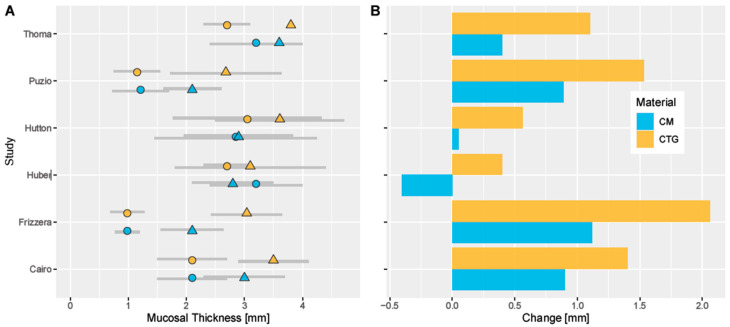
Left side (**A**): Average thickness of mucosa at baseline (circles) and at the end of the observation period (triangles) for different augmentation materials. Error bars indicate one standard deviation. Right side (**B**): Absolute change of mucosal thickness between baseline and end of observation period. X-axis scales are not proportional. CM: Collagen matrix. CTG: Connective tissue graft.

The study characteristics and the authors’ conclusions are presented in Table 1. Different techniques were used to measure the thickness of oral mucosa: two authors measured with CAD/CAM (Computer Aided Design/Computer Aided Manufacturing) manufactured stents and endodontic needles [17,19], three just with endodontic needles [16,20,22], one with an ultrasonic device [18], and one with CBCT-scans with a small field of view [21] to ensure a high quality picture. The CAD/CAM-made stents had guide channels for the endodontic needles so that the measurements were particularly reproducible. All authors used fixed measurement points. The outcomes are illustrated in Figure 2 and presented in Table 2. The surgical procedures are presented in Table 3. The baseline was defined as the time of the first measurement before any augmentation took place, with the exception of the work of Huber et al. and Thoma et al [20,22]. 2020, where the baseline was the insertion of the prosthetic crowns.

Cairo et al. [16] reported 2.1 mm (SD ± 0.6) mucosal thickness at baseline (second stage surgery of implantation) for both groups. After six months the mean thickness for the patients treated with CTGs rose to 3.5 mm (SD ± 0.6), the CM group showed 3 mm (SD ± 0.7), which was classified as a significant difference favoring the CTG. Hutton et al. [19] reported 3.05 mm (SD ± 1.28) at baseline (augmentation took place at the implant insertion) and 3.61 mm (SD ± 1.11) after four months for the CTG group. The acellular dermal matrices (ADMs) group started at 2.85 mm (SD ± 1.4) and ended with 2.9 mm (SD ± 0.94), showing no significant differences between the groups. Frizzera et al. [21] reported 0.98 mm (SD ± 0.29) for the CTG group and 0.98 mm (SD ± 0.21) for the CM group at baseline (augmentation performed during implant placement). After 12 months, the final thickness in the CTG group was 3.04 mm (SD ± 0.61) and 2.1 mm (SD ± 0.54) for the CM group, showing a significant difference towards the CTGs. As described in Table 1, Puzio et al. [18] had four different groups in the trial. They compared CMs with CTGs, once with augmentation three months before implantation (IIa and IIb) and once three months later, when the implant was uncovered (IIIa and IIIb). CMs three months prior to implantation (IIa) showed a mean thickness of 1.3 mm (SD ± 0.46), while CTGs (IIb) measurements demonstrated 1.30 mm (SD ± 0.23) at baseline. CMs three month after to implantation (IIIa) showed a mean thickness of 1.3 mm (SD ± 0.46), while CTGs (IIb) measurements displayed 1.30 mm (SD ± 0.23) at baseline. At the follow-up after twelve months, the soft tissue thickness of the individual groups presented as follows: IIa 2.46 mm (SD ± 0.75), IIb 3.06 mm (SD ± 0.61), IIIa 2.10 mm (SD ± 0.50) and IIIb 2.68 mm (SD ± 0.96). Thoma et al. [17] placed the grafts six weeks to six months after implantation. The CTG-Group presented a mean gain of 1.1 mm (SD ± 1.4 mm) while the CM-Group gained 0.8 mm (SD ± 2.2). Two follow-up studies were published for this trial. The first by Huber et al. [22] determined the soft tissue thickness 12 months after placement of the final restoration, which was roughly 15 months after surgery. CTGs at baseline was on average 2.7 mm (SD ± 0.4) thick, CMs showed a mean thickness of 3.2 mm (SD ± 0.8). Over the course of 15 months, CTGs gained 0.4 mm for an average of 3.1 mm (SD ± 1.3), while the CM-Group lost 0.4 mm, resulting in a mean thickness of 2.8 mm (SD ± 0.7). Thoma et al. [20] took another measurement after an additional 24 months, so three years after the restorations were inserted. Patient treated with CTGs showed a mean thickness of 3.8 mm (SD ± 1.5), while Thoma et al. [20] reported 3.6 mm (SD ± 1.5) for the CM-Group. The results are presented graphically in Figure 2 and in Table 2.

### 2.3. Patient-Reported Outcome Measurements (PROMs)

Five of the included studies [16,17,19,20,22] reported on patient-reported outcome measurements (PROMs). Two [16,19] used a 100-point VAS (visual analog scale) to assess postoperative discomfort and overall satisfaction. Thoma et al. [17] observed the amount of painkillers consumed. In addition, patients provided information on their perceived pain level after seven to 10, 30 and 90 days using a VAS questionnaire. They also filled out “oral health impact screening” questionnaires (OHIP-G14) [23] at screening date, seven to 10 days and 90 days after surgery. Huber et al [22]. used the same questionnaire at baseline, after 30 and after 90 days during their follow-up study.

The results are presented in Table 4. Cairo et al. [16] reported significantly better patient-reported outcomes for the CM group, with higher final satisfaction, less post-operative pain and lower painkiller intake post-surgery. Thoma et al. [17] concluded no significant differences between the two groups regarding OHIP, but patients in the CTG group reported having consumed more painkillers and showed higher VAS levels. At the time of suture removal, the CTG group had 100% higher pain scores than the CM group. Hutton et al. Huber et al. and Thoma et al. (2016) [17,19,22] presented no statistically significant differences between groups. Thoma et al. (2020) [20] reported statistically higher OHIP scores for the CM group.

### 2.4. Esthetic Outcomes

Only three of the studies included in this review [20,21,22] collected data on esthetic outcomes using the Pink Esthetic Score (PES) [24]. The PES consists of seven variables (e.g., soft tissue contour, mesial papilla, distal papilla etc.) and uses a scoring system ranging from 0 (lowest) to 2 (highest value) for each. Frizzera et al. [21] also utilized the modified Pink Esthetic Score (mPES) [25]. The authors of both studies used pictures taken with a periodontal probe held next to the area of interest at baseline, after six and after 12 months, which were evaluated and analyzed. The results are presented in Table 4.

Frizzera et al., Huber et al. and Thoma et al. [20,21,22] showed slightly higher PES and mPES scores for the CTG group, but these differences were not statistically significant.

### 2.5. Complications

Although explicitly mentioned only in three studies [17,18,21] as an outcome, we analyzed all articles for any reported complications during or after surgery. No implants or transplants failed, and only minor complications occurred. Hutton et al. [19] reported wound dehiscences for ten patients, seven of which occurred in the ADM-Group and three in the CTG group. According to the authors, these dehiscences were treated in the first four weeks and did not influence the final results. The authors of all studies concluded that soft tissue augmentation is a safe procedure, regardless of the materials used. Frizzera et al. [21] reported two patients in the CM group who developed an inflammation in the buccal area and an inflammation in the CTG group caused by a particle of the bone grafting material. The results are presented in Table 4.

### 2.6. Width of keratinized Mucosa

Five of the included studies reported the width of keratinized mucosa. The results are presented in Table 3. None of these studies showed significant differences between the groups.

### 2.7. Other Results Worth Mentioning

Cairo et al. [16] reported a mean surgical time of 35.5 min for augmentation using a CM versus 51.7 min when transplanting a CTG. Frizzera et al. [21] reported mesial and distal papilla migration and marginal peri-implant recessions. The papilla migration showed no statistically significant differences. While the control group (0.72 ± 0.52) and the CM-Group (0.42 ± 0.60) suffered marginal recessions after 12 months, the CTG group presented an elevated marginal ridge (−0.04 ± 0.3). The overall reported implant success rate was 100%. Thoma et al. and Cairo et al. [16,17] examined the peri-implant tissue health (BOP, probing depth). No statistical differences were reported.

All secondary outcomes are presented in Table 3 and Table 4.

### 2.8. Risk of Bias within Studies

Five of the included studies were classified as RCTs [16,17,18,19,21]. The risk of bias assessment is presented in detail in the additional materials and simplified in Table 5. Three authors were contacted for more information, because the study protocols were not publicly available. Only one author [18] replied and provided information in form of an internal protocol.

Three of the included studies were subject to a high risk of bias for one or more key domains [16,17,21]. Two studies showed an unclear risk of bias for one or more key domains [17,19]. Only one of the included studies was classified as “low risk” of bias in all domains [18].

### 2.9. Sources of Funding

None of the included studies reported any conflict of interest. Cairo et al., Thoma et al., Huber et al. and Puzio et al. received funding from Geistlich Pharma AG, Wolhusen, Switzerland. Hutton et al. was supported with an unrestricted grant from BioHorizons Inc., Birmingham, AL, USA. Frizzera et al. received funding in form of materials from Geistlich Pharma AG, Wolhusen, Switzerland and Conexoa Sistemas de Prótese. All authors point out that the financing of studies did not influence the results.

## 3. Discussion

### 3.1. Summary of Evidence

The question we addressed with this systematic review is: “Can substitute materials provide similar results to the subepithelial connective tissue graft for soft tissue augmentation around dental implants?” We focused on soft tissue thickness as the outcome, because it is an important factor regarding peri-implant health [11,26,27,28] as well as esthetic results [24]. Although the protocols of the included RCTs were heterogeneous (different augmentation times, follow-ups, materials used etc.), it can be asserted that the both use of substitute materials and connective tissue grafts lead to a thickening of the peri-implant oral mucosa to a similar extent. These results confirm prior pilot studies [29,30]. Thoma et al. and Huber et al. even reported slightly thicker mucosal tissue for patients treated with a volume stable collagen matrix in comparison to CTGs, although not statistically relevant. It should be noted that the graft used by Hutton et al., which was also comparable to CTGs in terms of increasing oral mucosa thickness, is not yet authorized for use by European practitioners (Alloderm, BioHorizons). In none of the included studies did the xenogeneic collagen matrices (XCMs) or ADMs prove to be superior to the gold standard, the connective tissue graft. While in four studies [17,18,19,22] no significant difference could be shown Cairo et al. and Frizzera et al. [16,21] found significant differences favoring the CTG over XCMs.

Soft tissue augmentation has become standard care in conjunction with implants in the esthetic zone [31] and in immediate implant placement to stop or at least slow down dimensional alterations which can occur [26]. However, with regard to postoperative morbidity after these kinds of procedures, biomaterials have been found to increase patients’ comfort and acceptance. The Patient-Reported Outcome Measurements indicate that substitute materials could be an alternative for pain-sensitive patients as a second surgical area is not required. Other benefiting patient groups could be those who have a higher risk of post-surgical complications, for example, wound healing problems in diabetics, especially if the graft’s removal technique requires secondary wound healing or an increased risk of bleeding in patients who take anticoagulants.

If we take into account the possible risks of harvesting a soft tissue graft from the patient’s palate, and the difficulty of performing this procedure, the use of substitute materials may be preferred by surgeons who want to avoid the procedure of CTG removal and; therefore, lead to a higher acceptance of soft tissue thickening procedures from the clinician’s perspective. However, the authors explicitly point out that any operations to thicken soft tissue around implants, whether using CTGs or substitute materials, should only be performed by experienced and highly skilled practitioners. However, a similarity to bone augmentation cannot be denied. The harvesting of autologous bone, especially larger quantities or bone blocks, can be considered technically demanding and time-consuming. Since the introduction of bone replacement materials on the market, whether allogenic, xenogeneic or alloplastic, the acceptance of this treatment method among practitioners has increased. Bone augmentation before or during implantation is now part of standard care.

Peri-implant soft-tissue thickness has an impact on the shine-through of dental restorative materials [32]. Increased mucosal thickness could lead to lower shine-through effects and improve the aesthetic result, which can be seen as one of the most, or the most, important factors for the patient. Jung et al. were able to demonstrate in their in vitro experiment that there were no more translucency effects at a mucosa thickness of 3mm and this independent of the restoration material used. Cairo et al. [16] concluded that 79% of XCM- and 93% of CTG-treated sites achieved final soft tissue thickness ≥2.5 mm, which classifies as thick biotype [1]. Puzio et al. [18] surpassed these numbers and presented a thick biotype in all groups.

An interesting and for the practitioner possibly important aspect are the different ways to measure mucosal thickness and gingival biotype, which are seen as key determinants for treatment planning by some authors [33]. According to a well-known study [34], the gingival biotype itself can be reproducibly determined by visual assessment. In contrast to these findings, another study concluded that “simple visual inspection may not be considered a valuable method to identify the gingival biotype as nearly half of the high-risk patients are overlooked” [35]. This statement is supported by the results of Puzio et al. [18], who showed that ultrasound measurement led to a different biotype classification than measurement with a periodontal probe. CBCT scans, which are standard care for many surgeons in esthetically challenging areas, can be an alternative for preoperative thickness assessment. Although, in the opinion of the authors of this article, the measurement of soft tissue alone does not justify the resulting radiation exposure. However, if preoperative CBCT scans are performed before, for example, implantation, these data sets can be used to determine soft tissue thickness. A recent study [36] compared four different techniques to determine soft tissue thickness. Transgingival probing with a periodontal probe, transgingival probing with a stainless steel acupuncture needle and use of an ultrasonic device and a color-coded periodontal probe. The authors of the study conclude that the measurement with the periodontal probe and the ultrasound device provided the most reproducible results. The disadvantages of measurement with the periodontal probe are invasiveness, the need to eliminate pain and the lower accuracy. According to Slak et al. [37], ultrasound devices can measure the thickness of the gingiva to within two decimal places, whereas the periodontal probe only has a millimeter scale.

Another point that is unfortunately rarely mentioned but should not be forgotten is the origin of the biomaterials. The known xenogeneic matrices have a porcine origin and; therefore, for many people, for religious reasons, do not represent an alternative to CTGs.

### 3.2. Agreements and Disagreements with Previous Studies

In our opinion the meta-analysis of Gargallo-Albiol et al. [15] offers several points worthy of discussion. Methodologically, the first point that stands out is the very low number of matches (*n* = 133) in the systematic search. A precise and narrow search query has the advantage of reducing the work required for screening, but as limited retrieval might be not comprehensive studies can easily be overlooked. A study published in 2017 [21], was included in this review; however, was not included in the review mentioned above for unclear reasons. Gargolla-Albiol et al. [15] considered two studies [21,22] as low risk of bias, which were clearly defined as high risk in our review in accordance with the Cochrane Collaboration’s tool [38] criteria, since no study protocols were available, and in the case of Thoma et al. [17], no sufficient information on the sequence generation process was provided. A main difference between the two reviews concerns the representation of the findings of Cairo et al. [21]: The above-mentioned review states, as Cairo’s conclusion, that there is no difference in gingival thickness between the two groups of the trial. However, if we look at the original paper, we see that Cairo presents a significant difference in favor of the CTG. In addition, Gargallo-Albiol et al. included a study of Sanz et al. [39], but did not recognize that not only was soft tissue grafted around implants, but also natural teeth were part of both groups (*n* = 6 of 20 sites in total). For this same reason and the lack of gingival thickness as an outcome parameter, we have not included the trial. The review under discussion included a study by Zeltner et al. [40]. However, it is not clearly stated that Zeltner performed his measurements on the patient pool of Thoma et al. [21], only the study of Huber et al. is mentioned. Zeltner et al. [40] determined the volumetric changes in a region of interest using scanned plaster models. The impressions were taken at the same time as Thoma et al. measured. Since Thoma already reported on the thickness of the gingiva as an outcome, we saw no reason to include Zeltner’s data. Nevertheless, we believe that this fact should have been presented more explicitly by Gargallo-Albiol. We classified Huber et al. [22] and Thoma et al. 2020 [20] as follow-up studies, because the intervention was part of another trial and only new measurements were performed. Finally, we would like to point out again that, in our opinion, a statistical meta-analysis is not appropriate due to the wide heterogeneity of study designs. As a result, our conclusion differs from the previously discussed meta-analysis.

### 3.3. Limitations

The main limitation of this systematic review was the heterogeneity and the small number of included studies. Due to the different methodical approaches used, we are not able to make any definitive statements regarding the superiority of CTGs over alternative materials which are universally applicable. In addition, a clear statement is made more difficult by the increased risk of bias of the individual studies. Only one study fulfilled the criteria for low risk of bias. Defective study designs that result, for example, in an inadequate allocation concealment should not be ignored. The influence of these errors on the overall result cannot; however, be determined by the authors of this review.

## 4. Materials and Methods

This systematic review was conducted according to the standards of the PRISMA (Preferred Reporting Items for Systematic Reviews and Meta Analyses) Statement [39], the Cochrane Handbook of Systematic Reviews of Interventions [41] and the AMSTAR 2 guidelines for systematic reviews that include randomized or nonrandomized studies of healthcare interventions [42].

### 4.1. Eligibility Criteria

The following search and eligibility criteria were defined according to PICO(S) analysis [43], which allowed us to convert our clinical findings and problems in a clearly defined question:

P—Population

I—intervention

C—Comparison

O—Outcome

The population was defined as patients receiving a soft tissue augmentation during implantation or for existing dental implants. The intervention was a soft tissue augmentation using a substitute material (e.g., xenogeneic collagen matrix or dermal allografts). The control group was treated with a subepithelial connective tissue graft. The observed primary outcome was volume gain in mucosal tissue around the dental implant, and secondary outcomes were PROMs (Patient Reported Outcome Measurements), esthetic outcomes and complication rates. Eligible studies were randomized controlled clinical trials with at least 3-month follow-up duration and at least 10 participants per group.

### 4.2. Information Sources and Search

Two investigators (MB and ML) searched the electronic databases MEDLINE via OvidSB and all included databases in the Web of Science for relevant publications from 1995 on 20 June 2020. In addition, we hand-searched previous reviews on related topics. The search string comprised a combination of key words (MeSH) and free-text terms and was designed eligibility criteria. The search expression comprised three subexpressions: (1) On the dental implant, (2) on the transplant, and (3) on the main outcome (thickness of oral mucosa).

### 4.3. Study Selection and Data Extraction

Duplicate references were eliminated from the combined result set. Inclusion and exclusion criteria were derived from the eligibility criteria. Two investigators (AH and ML) reviewed the titles and abstracts independently and agreed on selection of studies that fit the predetermined inclusion criteria. Full-text articles were reviewed for included studies and for studies which could not be excluded based on title and abstract. The two investigators achieved consensus through discussion on which studies to include.

#### 4.3.1. The Following Inclusion Criteria Were Applied

- Publication in the peer-reviewed literature;
- Full text available in English or German;
- (Randomized) controlled clinical trials;
- Investigated soft tissue grafting during implantation or around existing dental implants using subepithelial connective tissue grafts and substitute materials;
- Reported on soft tissue thickness;
- Follow-up of at least three months.

#### 4.3.2. The Following Exclusion Criteria Were Applied

- Animal studies;
- In-Vitro studies.

The restrictions for inclusion are justified as followed: Only peer-reviewed literature was adopted to ensure that all studies meet high scientific and current standards. The authors of this review speak fluent German and English, publications in other languages would have made a detailed review far more difficult. In order to obtain a meaningful comparison of substitute materials and CTGs, randomized controlled trials were preferred. An observation period of three months was established as a minimum to detect medium-term changes in soft tissue thickness and to distinguish it from short-term results.

### 4.4. The following Items Were Extracted from the Included Studies

Author; Year of publication; Source; Country of origin; Assessed outcomes; Follow-up time; Sample size; Age of the population; Group distribution; Inclusion and exclusion criteria; Drop-outs; Complications; Method of surgery; Occurrence of bone augmentation; Materials used; Regions of implantation; Number of implants; Time of soft tissue augmentation; Consideration of the biotype; Post-implantation behavior; Prosthetic restoration of the implant; Method of outcome assessment; Data analysis; Findings.

### 4.5. Synthesis of Results

Relevant data was collected and summarized in tables and graphics. A narrative summary was created for each study. Due to the heterogeneity between the studies regarding materials used, time of augmentation, observation periods, etc., no meta-analysis could be performed.

### 4.6. Risk of Bias Assessment

Two authors (MB and ML) independently assessed the risk of bias for the included studies with the Cochrane Collaboration’s tool [38] and resolved all conflicting judgements through discussion.

## 5. Conclusions

This systematic review is the second to assess the effectiveness of acellular dermal matrices and xenogenic collagen matrices in comparison to connective tissue grafts for the augmentation of oral mucosa around dental implants with long term results over a period up to three years. From an initial search result set of 1050 references, seven articles were included in this review. The methodical quality of the included studies was low overall: Only one study showed low risk of bias in all key domains. Characteristics of the studies were very heterogeneous, so no quantitative synthesis could be performed. Both the CTGs and the substitute materials resulted in increased mucosal thickness. Five studies showed no significant difference, while two presented a significant difference favoring the CTGs over alternative materials.

Soft tissue augmentation around dental implants is a safe procedure and leads to thicker mucosal tissue. The subepithelial connective tissue graft can still be regarded as the gold standard, but substitute materials may be an alternative for sites where only minor thickening is needed, for patients who are pain-sensitive or for patients who do not consent to harvesting from the palate or simply do not have enough tissue at the palate. Moreover, these materials also may be an alternative for dentists who are not trained to, or comfortable with, harvesting connective tissue grafts.

## Figures and Tables

**Table 1:** Study characteristics and authors’ conclusions. IG: Intervention Group. CG: Control Group. CM: Collagen matrix. CTG: Connective tissue graft. PROMS: Patient reported outcome measurements.

	Type of Study	Groups and Transplants Used	Time of Augmentation	No. of Patients/Sites	Follow-Up (Months)	No. of Patients/Sites	Reported Outcomes	Age of Participants	Smokers Accepted	Author’s Conclusion
Cairo et al. 2018 [16]	RCT	IG:Xenogeneic collagen matrix (Mucograft, Geistlich) vs. CG:Connective tissue graft	Second stage surgery (No further information)	60/60	6	60/60	1. Changes of mucosal thickness 2. Width of keratinized gingiva 3. PROMs 4. Surgery time	CM 50.3 ± 12.4 CTG 48.3 ± 11.8	Yes (<10 cigarettes per day)	Significant difference favouring CGT
Hutton et al. 2018 [19]	RCT	IG: Allograft (Alloderm, BioHorizons) vs. CG: Connective tissue graft	During implant surgery	20/20	4	20/20	1. Changes of mucosal thickness 2. Width of keratinized gingiva 3. PROMs 4. Modified wound healing index	55.5 ± 11.5	Not accepted	No significant difference
Frizzera et al. 2018 [20]	RCT	Xenogeneic collagen matrix (Mucograft, Geistlich) vs. Connective tissue graft vs. No graft	During IIPP-Surgery (Immediate Implant Placement and Provisionalisation)	24/24	12	24/24	1. Marginal periimplant recession after IIPP 2. Changes of mucosal thickness 3. PES and mPES 4. Facial bone thickness 5. Papilla migration 6. Periodontal health 7. Implant Success-rate	23–65	Not accepted	Significant difference favouring CGT
Puzio et al. 2018 [18]	RCT	See table (/)	3 months prior or 3 months after implant placement	57/75	12	57/75	1. Changes of mucosal thickness 2. Biotype 3. Implant Success-rate	18–60	Yes (<10 cigarettes per day)	Significant difference favouring CGT
Thoma et al. 2016 [17]	RCT	IG: Xenogeneic collagen matrix (Fibro-Gide, Geistlich) vs. CG: Connective tissue graft	6 weeks to 6 months after implant placement	20/20	3	20/20	1. Changes of mucosal thickness 2. Width of keratinized gingiva 3. PROMs 4. Histological evaluation 5. Periodontal health 6. Safety evaluations	CM 43.8 ± 13.2 CTG 42.7 ± 19.1	Yes (<10 cigarettes per day)	No significant difference
Huber et al. 2018 [22] Thoma et al. 2020 [20]	FU	See Thoma et al. [20]	See Thoma et al. [20]	20/20 17/17	12 (after insertion of final restoration → 15 months after surgery) 36 months	20/20	1. Changes of mucosal thickness 2. PES and PES 3. Width of keratinized gingiva 4. Periodontal health 5. PROMs	See Thoma et al. [20]	See Thoma et al. [20]	No significant difference

**Table 2:** Soft tissue thickness. CM: Collagen matrix. CTG: Connective tissue graft. ADM: Acellular dermal matrix. BL: Baseline. FU: Follow-Up.

	Time of Augmentation	Follow-Up (Months)	Measurement- Technique for Soft Tissue Thickness	Outcome Soft Tissue Thickness (mm)							Change in ST-Thickness (mm) BL–Last FU							
Cairo et al. 2018 [16]	Second stage surgery (No further information)	6	Endodontic needle (1mm coronal to mucogingival junction)				CM			CTG	CM 0.9			CTG 1.4				
				Baseline			2.1 ±0.6			2.1 ±0.6								
				3 months			2.8 ±0.7			3.1 ±0.5								
				6 months			3.0 ±0.7			3.5 ±0.6								
Hutton et al. 2018 [19]	During implant surgery	4	CAD/CAM produced stent with 3 measurement points (1, 3 and 5 mm [B1-B3] apical from the mucosal margin) and endodontic needle			CTG B1	CTG B2			CTG B3	CTG B1			CTG B2			CTG B3	
				Baseline		3.05 ± 1.28	2.95 ± 1.17			1.65 ± 0.75	0.44 ± 2.04			1.2 ± 1.48			1.2 ± 0.89	
				Final		3.61 ± 1.11	4.15 ± 1.33			2.85 ± 0.58								
						ADM B1	ADM B2			ADM B3	ADM B1			ADM B2			ADM B3	
				Baseline		2.85 ± 1.40	2.40 ± 1.02			1.70 ± 0.67	0.05 ± 1.57			0.85 ± 1.29			1.45 ± 1.17	
				Final		2.90 ± 0.94	3.25 ± 1.30			3.15 ± 0.94								
Frizzera et al. 2018 [21]	During IIPP-Surgery (Immediate Implant Placement and Provisionalisation)	12	CBCT with a small field of view (2mm below the gingival margin)			Control	CM			CTG	Control 1.11			CM 1.12			CTG 2.06	
				Baseline		1 ± 0.18	0.98 ± 0.21			0.98 ± 0.29								
				6 months		2.04 ± 0.43	2.05 ± 0.41			2.82 ± 0.40								
				12 months		2.11 ± 0.60	2.10 ± 0.54			3.04 ± 0.61								
Puzio et al. 2018 [18]	3 months prior (II) or 3 months after (III) implant placement a = CM b= CTG	12	Ultrasonic device (Pirop®, Echoson) (Point 1: on the line connecting the two cemento-enamel junctions of both adjectent teeth; Point 2: On the mucogingival junction			I	IIa	IIb	IIIa	IIIb		I		IIa	IIb		IIIa	IIIb
				Point 1	BL	1.39 ± 0.65	1.30 ± 0.46	1.30 ± 0.23	1.21 ± 0.49	1.15 ± 0.40	Point 1	0.7 ± 0.8		1.16 ± 0.7	1.76 ± 0.7		0.89 ± 0.6	1.52 ± 1.0
				Point 2	BL	1.10 ± 0.44	1.04 ± 0.47	0.75 ± 0.26	1.01 ± 0.41	0.90 ± 0.30								
				Point 1	12 m	2.10 ± 0.66	2.46 ± 0.75	3.06 ± 0.61	2.10 ± 0.50	2.68 ± 0.96	Point 2	0.35 ± 0.6		1.0 ± 0.7	1.36 ± 0.6		0.57 ± 0.6	1.15 ± 0.5
				Point 2	12 m	1.46 ± 0.34	2.04 ± 0.61	2.11 ± 0.70	1.57 ± 0.52	2.05 ± 0.56								
Thoma et al. 2016 [17]	6 weeks to 6 months after implant placement	3	CAD/CAM produced stent and endodontic needle 3 points of measurement (occlusal, buccal and apical)	Baseline														
						CM			CTG		CM					CTG		
				Occlusal		3.4 ± 1.0			4.2 ± 1.9		1.4 ± 1.4					0.8 ± 1.8		
				Buccal		2.9 ± 1.5			4.1 ± 2.0		1.1 ± 1.4					0.8 ± 2.2		
				Apical		2.6 ± 2.3			3.4 ± 1.8		0.9 ± 1.9					1.6 ± 2.6		
				FU-90														
				Occlusal		4.25			4.0									
				Buccal		4.0			5.3									
				Appical		2.5			5.0									
Huber et al. 2018 [22] Thoma et al. 2020 [20]	See Thoma et al. [17]	12 (after insertion of final restoration) /36 months	Endodontic needle (1mm apical of the margo mucosae)			CM			CTG						CM			CTG
				Baseline		3.2 ± 0.8			2.7 ± 0.4		BL		6 months		–0.3 ± 0.9			0.3 ± 1.0
				6 months		2.9 ± 0.9			3.0 ± 0.9		BL		12 months		–0.4 ± 0.9			0.4 ± 1.4
				12 months		2.8 ± 0.7			3.1 ± 1.3		BL		36 months		0.44 ± 1.1			1.1 ± 1.5
				36 months		3.6 ± 1.5			3.8 ± 1.5									

**Table 3:** Secondary Outcomes I. CM: Collagen matrix. CTG: Connective tissue graft. FGG: Free gingival graft. ADM: Acellular dermal matrix. CTL: Control. BOP: Bleeding on probing. PPD: Pocket probing depth.

	Surgical Technique	Width of Keratinized Gingiva (mm)			Surgery Time (min)	Initial Phenotype		Periimplant Tissue Health (BOP, PPD)
Cairo et al. 2018 [16]	Preparation of split-thickness-flap.In the test group, first a collagen matrix was secured supraperiosteally, after which a second matrix was applied over the first. The matrices were sutured to the periosteum (absorbable sutures). Thus the total thickness was 6mm. In the control group, the connective tissue transplants were harvested from the palate via trap-door approach or as deepithialized FGG and sutured to the periosteum. The thickness was about 1mm throughout.		CTG	CM	CTG 51.7 ± 7 CM 35.5 ± 9.4	Not reported		No statistically significant differences
		Baseline	3.5 ± 1.7	3.1 ± 1.2				
		Final	4.4 ± 1.5	4.3 ± 1.2				
Hutton et al. 2018 [19]	A combination of full thickness and partial thickness flap was prepared as the recipient bed for the graft. In the control group, a connective tissue graft was taken from the palate. The ADM graft of the test group was adapted and processed according to the manufacturer’s instructions, taking care to ensure that the dimensions were similar to those of the control group.		CTG	ADM	Not reported	Not reported		Not reported
		Baseline	5.30 ± 1.16	4.95 ± 1.38				
		Final	4.45 ± 1.14	4.50 ± 0.94				
		Change	–0.85 ± 1.13	–0.45 ± 1.30				
Frizzera et al. 2018 [21]	In the CM and CTG group, the buccal mucosa was undermined and a pocket was prepared without damaging the papillae. The height of the grafts was always 6mm, the length was determined by the distance between the mesial and distal papilla. In the CTG group, a 1.5mm thick palatal mucosal graft was harvested and the epithelial portion was removed with a 15C blade. In the CM group, the graft was trimmed according to the specifications.The grafts were sutured to the gingival margin. In addition, all bone defects were covered with a membrane (Bio-Gide, Geistlich) and the space between implant and membrane was filled with Bio-Oss Collagen (Geistlich).	Not reported			Not reported		Thin/Thick	Not reported
						CTG	5/3	
						CM	4/4	
						CTL	5/3	
Puzio et al. 2018 [18]	The recipient bed was prepared as a mucosa flap ("envelope technique"). The roots of the adjacent teeth were smoothed with a fine diamond and the adjacent papillae were deepithelialized. The BGT was removed from the palate using the single-incision technique. The CMX graft was processed according to the manufacturer’s instructions. For suturing, the flap was placed slightly above the CEJ.	Not reported			Not reported	All patients presented a thin biotype		Not reported
Thoma et al. 2016 [17]	A mucoperiosteal flap (full thickness flap) was prepared, which was split at the border to the buccal bone portion (partial thickness flap). A pocket was then formed buccally to receive the graft and buccal relief incisions were made to allow tension-free wound closure. In the test group the collagen matrix graft was cut accordingly. In the control group the graft was removed from the palate using the single incision technique. The grafts were placed in the prepared pockets and secured with sutures, then the wound was closed.	Data not shown, but no statistically significant differences reported at the target site.			Not reported	Not reported		Data not shown, but no statistically significant differences reported at the target site.
Huber et al. 2018 [22]	See Thoma et al. [17]		CTG	CM	Not reported	Not reported		No statistically significant differences
		Baseline	3.2 ± 1.4	2.5 ± 0.8				
		Final	3.2 ± 0.8	2.1 ± 1.2				
		Change	0.0 ± 1.2	–0.2 ± 0.7				
Thoma et al. 2020 [20]	See Thoma et al. [17]	No statistically significant differences			Not reported	Not reported		No statistically significant differences

**Table 4:** Secondary Outcomes II. PROMS: Patient reported outcome measurements. PES: Pink Esthetic Score. mPES: Modified pink esthetic score. CM: Collagen matrix. CTG: Connective tissue graft. VAS: Visual analog scale. OHIP: Oral health impact.

	Assessment of PROMs	Outcomes of PROMs			Complications	Implant Success Rate	Esthetic Evaluation	PES/mPES-Scores					
Cairo et al. 2018 [16]	100-point VAS (Visual Analog Scale) to assess postoperative discomfort and overall satisfaction	CTG: 35 ± 23 CM: 17 ± 13 Patients in the CM group experienced significantly less postoperative pain (13.0 ± 10 vs. 37.0 ± 15; *p* < 0.0001), consumed less anti-inflammatory medication (2.2 ± 0.8 vs. 3.9 ± 0.7; p < .0001), and fewer uncomfortable days (1.2 ± 0.7 vs. 2.4 ± 0.7; *p* < 0.0001). No significant differences regarding aesthetic outcome.			1mm soft tissue recession of one patient in the CTG group.	100%	Not reported	-					
Hutton et al. 2018 [19]	100-point VAS (Visual Analog Scale) to assess postoperative discomfort and overall satisfaction	Discomfort (1–100)			Three patients in the control group and seven patients in the experimental group showed postoperative wound dehiscence, which was treated within the first 4 weeks and, according to the authors, did not influence the final result.	100%	Not reported	-					
			CTG	ADM									
		2 weeks	23.60 ± 24.71	10.10 ± 7.78									
		4 weeks	10.40 ± 16.51	4.40 ± 4.25									
		8 weeks	9.70 ± 15.54	4.40 ± 7.99									
		16 weeks	7.50 ± 15.48	6.70 ± 9.53									
		Overall Satisfaction (1–100)											
			CTG	ADM									
			98.30 ± 2.26	94.80 ± 7.31									
Frizzera et al. 2018 [21]	Not reported	-			One patient in the CTG group lost the temporary crown after 4 months. Two patients of the CM group showed inflammation of the facial peri-implant tissue. One particle of the bone grafting material caused soft tissue inflammation in one patient of the CTL group.	100%	PES and mPES at baseline and after 6 month and 12 months	PES	Control		CM		CTG
								Baseline	10.75 (2.05)		10.63 (1.84)		9.37 (1.9)
								12 months	9.87 (1.64)		10 (1.3)		10.75 (1.38)
								mPES	Control		CM		CTG
								Baseline	7.00 (1.73)		7.75 (0.70)		7.00 (1.41)
								12 months	6.62 (1.59)		7.12 (0.99)		7.87 (0.99)
Puzio et al. 2018 [18]	Not reported	-			No complications reported.	100%	Not reported	-					
Thoma et al. 2016 [17]	Amount of painkillers consumed; VAS for postoperative discomfort, OHIP	Patients in the CTG group reported having consumed more painkillers and showed higher VAS levels. At the time of suture removal, the CTG group had 100% higher pain scores than the CM group.			In both groups, one treatment was classified as unsuccessful because no increase in volume was observed.	100%	Not reported	-					
Huber et al. 2018 [22]	OHIP	The average score for the OHIP questionnaire for both groups was 0 consistently.			See Thoma et al. [17]	100%	PES at baseline and after 6 months and 12 months	PES					
									CM			CTG	
								Baseline	9.6 ± 1.6			8.4 ± 3.5	
								6 month	8.8 ± 1.8			9.8 ± 3.3	
								12 month	8.9 ± 2.4			9.1 ± 2.1	
Thoma et al. 2020 [20]	OHIP	CM 0.5 CTG 0.0 Significant difference			See Thoma et al. [17]	100%	PES at 36 months	CM		CTG			
								8.5		10			

**Table 5:** RoB-Assesement.

	Hutton 2018	Cairo 2018	Frizzera 2018	Puzio 2018	Thoma 2016
Random sequence allocation	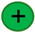	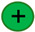	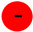	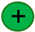	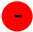
Allocation concealment	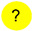	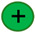	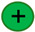	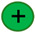	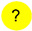
Blinding of participants and personnel	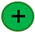	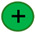	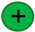	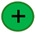	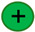
Blinding of outcome assessment	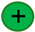	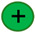	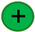	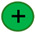	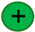
Incomplete outcome data	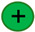	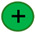	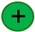	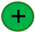	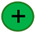
Selective reporting	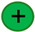	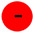	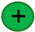	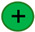	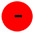

## Abbreviations

CM	Collagen matrix
ADM	Acellular dermal matrix
CTG	Connective tissue graft
